# Feasibility, safety, and long-term efficacy of gastric peroral endoscopic myotomy (G-POEM) for postsurgical gastroparesis: a single-center and retrospective study of a prospective database

**DOI:** 10.1007/s00464-020-07793-0

**Published:** 2020-09-03

**Authors:** Jiacheng Tan, Sachin Mulmi Shrestha, Ming Wei, Panpan Wang, Jinjun Shi, Yanjia Lu, Qi Gao, Tong Lu, Jun Zhou, Ruihua Shi

**Affiliations:** 1 Department of Gastroenterology, Zhongda Hospital Southeast University, No. 87 Dingjiaqiao, Nanjing, 210009 Jiangsu China; 2 Department of Ultrasound, Zhongda Hospital Southeast University, Nanjing, 210009 Jiangsu China; 3 Department of Radiology, Zhongda Hospital Southeast University, Nanjing, 210009 Jiangsu China; 4 Medical Division, Yidu Cloud (Beijing) Technology Co., Ltd, Beijing, 100191 China

**Keywords:** G-POEM, Postsurgical gastroparesis, GCSI, GERDQ, The anastomotic site, Gastric emptying

## Abstract

**Background:**

Postsurgical gastroparesis is recognized as a gastrointestinal dysfunction syndrome following foregut surgery. Gastric peroral endoscopic myotomy (G-POEM) is suggested as a minimally invasive therapy for gastroparesis. But the long-term efficacy and safety of G-POEM in treating postsurgical gastroparesis are rarely explored.

**Methods:**

The primary outcomes included the symptomatic improvement based on gastroparesis cardinal symptoms index (GCSI) and the improvement of gastric emptying. The secondary outcomes included the improvement of gastroesophageal reflux symptoms and complications of G-POEM.

**Results:**

The severity of postsurgical gastroparesis was not associated with the onset time and the course of the disease. G-POEM significantly reduced GCSI throughout the follow-up period (*p* < 0.0001). For different anastomotic site, a significant improvement of GCSI was found at 6 month post-G-POEM (*F*_4,165_ = 74.18, *p* < 0.0001). Subscale analysis of GCSI showed that nausea/vomiting, post-prandial fullness/early satiety, and bloating were improved significantly at 6-month post-G-POEM (*p* < 0.0001, respectively). Half-emptying and whole-emptying time were significantly shortened in patients with different anastomotic site post-G-POEM (half-emptying time: *F*_3,174_ = 65.44, *p* < 0.0001; whole-emptying time: *F*_3,174_ = 54.85, *p* < 0.0001). The emptying of ioversol was obviously accelerated after G-POEM. GCSI wasn't related to pyloric length, pyloric diameter, and thickness of pyloric wall. GERDQ was also used to evaluate the clinical efficacy of G-POEM. For each time points, GERDQ didn't differ significantly in patients with different anastomotic site (*F*_4,104_ = 0.8075, *p* = 0.5231). For patients with different anastomotic site, GERDQ was improved significantly at different time points (*F*_4,104_ = 59.11, *p* < 0.0001). The higher the esophageal anastomotic site was, the faster G-POEM improved the symptoms of gastroesophageal reflux. No one required re-hospitalization for any complication.

**Conclusion:**

G-POEM is a minimally invasive therapy with long-term effectiveness and safety in treating postsurgical gastroparesis.

**Electronic supplementary material:**

The online version of this article (10.1007/s00464-020-07793-0) contains supplementary material, which is available to authorized users.

Postsurgical gastroparesis refers to the gastrointestinal dysfunction syndrome that occurs after esophageal and gastrointestinal surgery and other surgical operations affecting the upper digestive tract. Postsurgical gastroparesis accounts for about 13% of gastric emptying disorder and is mainly characterized by non-mechanical obstruction of the gastric outflow tract [1]. The main clinical manifestations of postsurgical gastroparesis were nausea, vomiting, bloating, upper abdominal fullness, gastric retention, and weight loss, resulting in a poor quality of life.

In the past, few effective treatment existed. Conventional medical treatments (including life conditioning, gastrointestinal motility drugs, traditional Chinese medicine, acupuncture, gastric electrical pacing) usually failed to relieve symptoms in many patients [2]. Laparoscopic pyloroplasty or pyloromyotomy can improve the symptoms and gastric emptying function of postsurgical gastroparesis [3–5]. In order to make the operations more minimally invasive, endoscopic therapies (transpyloric stenting and botulinum toxin injection) emerged. However, both failed to improve gastroparesis symptoms when compared to placebo [6–9]. In a clinical guideline on the management of gastroparesis, intrapyloric injection of botulinum toxin is not recommended for patients with gastroparesis [2].

In recent years, peroral endoscopic myotomy (POEM) had been successfully applicated in the treatment of achalasia. Based on the theory of POEM, gastric peroral endoscopic myotomy (G-POEM) was gradually performed in the therapy of gastroparesis [10–17]. Nevertheless, there was few study about G-POEM in the therapy of postsurgical gastroparesis. Our experience in treating postsurgical gastroparesis with G-POEM was reported in the present study.

## Methods

This is a single-center and retrospective study of a prospective database performed on patients with postsurgical gastroparesis who underwent G-POEM between October 2016 and March 2019. Patients went to the GI outpatient and then admitted to the ward. The study was complied with the ethical requirements of the declaration of Helsinki and approved by our institutional research ethics board (Approval Number: ZDYJLY(2016)65). All the patients had signed the specific written informed consent prior to the procedure.

Inclusion criteria were as follows: 1. Patients aged 18 or older, with history of proximal gastrectomy and other surgical operations affecting the upper digestive tract. 2. Patients with clinical manifestations included nausea, vomiting, bloating, upper abdominal fullness, gastric retention, and weight loss, with gastroparesis cardinal symptoms index (GCSI) > 2.3 points. 3. Patients failed in conservative therapy (i.e., symptoms persisted during dietary modification and prokinetics/antiemetics treatment), and struggled with postsurgical gastroparesis for at least 6 months. 4. Patients with objective evidences of delayed gastric emptying: (1) Gastroscopy: Peristalsis diminished or disappeared in the residual stomach with gastric fluid retention, but no pyloric stenosis was found. (2) Upper gastrointestinal imaging: Peristalsis diminished or disappeared in the residual stomach. The contrast agent could not pass through the pylorus or could pass through the pylorus very slowly. Several hours later, large amounts of contrast agent remained in the residual stomach. However, no indication of pylorus obstruction was found. (3) Three-dimensional ultrasound: Peristalsis diminished or disappeared in the residual stomach. The pylorus opened as a thin line with occasionally contrast regurgitation. The half-emptying and whole-emptying time were significantly delayed.

Exclusion criteria were aas follows: 1. Patients with the mechanical obstruction of the gastric outflow tract. 2. Patients with the history of diabetes, hypothyroidism, connective tissue disease, neuromuscular disease, and other basic diseases that cause gastroparesis. 3. Patients with the history of other malignant tumors. 4. Patients who underwent additional pyloroplasty or pyloromyotomy in the disease duration of postsurgical gastroparesis. 5. Patients who could not tolerate general anesthesia or with any contraindication to an endoscopy. 6. Patients with pregnancy.

The primary outcomes included the symptomatic improvement based on GCSI and the improvement of gastric emptying. Clinical response was defined as more than 25% decrease in at least two subscales of the GCSI scale. The secondary outcomes included the improvement of gastroesophageal reflux symptom, complications of G-POEM, and other outcomes (e.g., BMI, diet status, and medications, and so on).

Medical management before the study was shown in Supplementary Table 1. All the patients were asked to stop using drugs that affect gastrointestinal smooth muscle contraction one week before G-POEM. All of them got the sufficient preoperative preparations, including symptom assessment, gastroscope, gastric emptying imaging, and three-dimensional ultrasound of gastric antrum. Symptoms of postsurgical gastroparesis were assessed by GCSI and GERDQ (Supplementary Table 2, Supplementary Table 3). The anastomosis sites were observed by gastroscope. The morphology of the residual stomach and the emptying of ioversol were observed by upper gastrointestinal imaging. The patient was asked to fast and abstain from water for 8 h, then take 200 ml iodine contrast agent and take X-ray immediately. Then, X-rays were taken at 30 min, 60 min, and 120 min. The volume of gastric antrum and the morphological structure of pylorus tube were observed by three-dimensional ultrasound. The half-emptying and whole-emptying time were calculated by analyzing the volume change of gastric antrum. Three-dimensional ultrasonography had been appeared to be an accurate and valid measurement of gastric emptying, which had good correlation and consistency with scintigraphy- the ‘gold standard’ technique to measure gastric emptying [18–23]. Follow-up data were obtained directly from the patients at 6-month, 12-month, 18-month, and 24-month post-G-POEM. The follow-up protocol is shown in Supplementary Table 4. Adverse events were graded according to the American Society for Gastrointestinal Endoscopy lexicon [24].

### G-POEM procedure

**Fig. 1:**
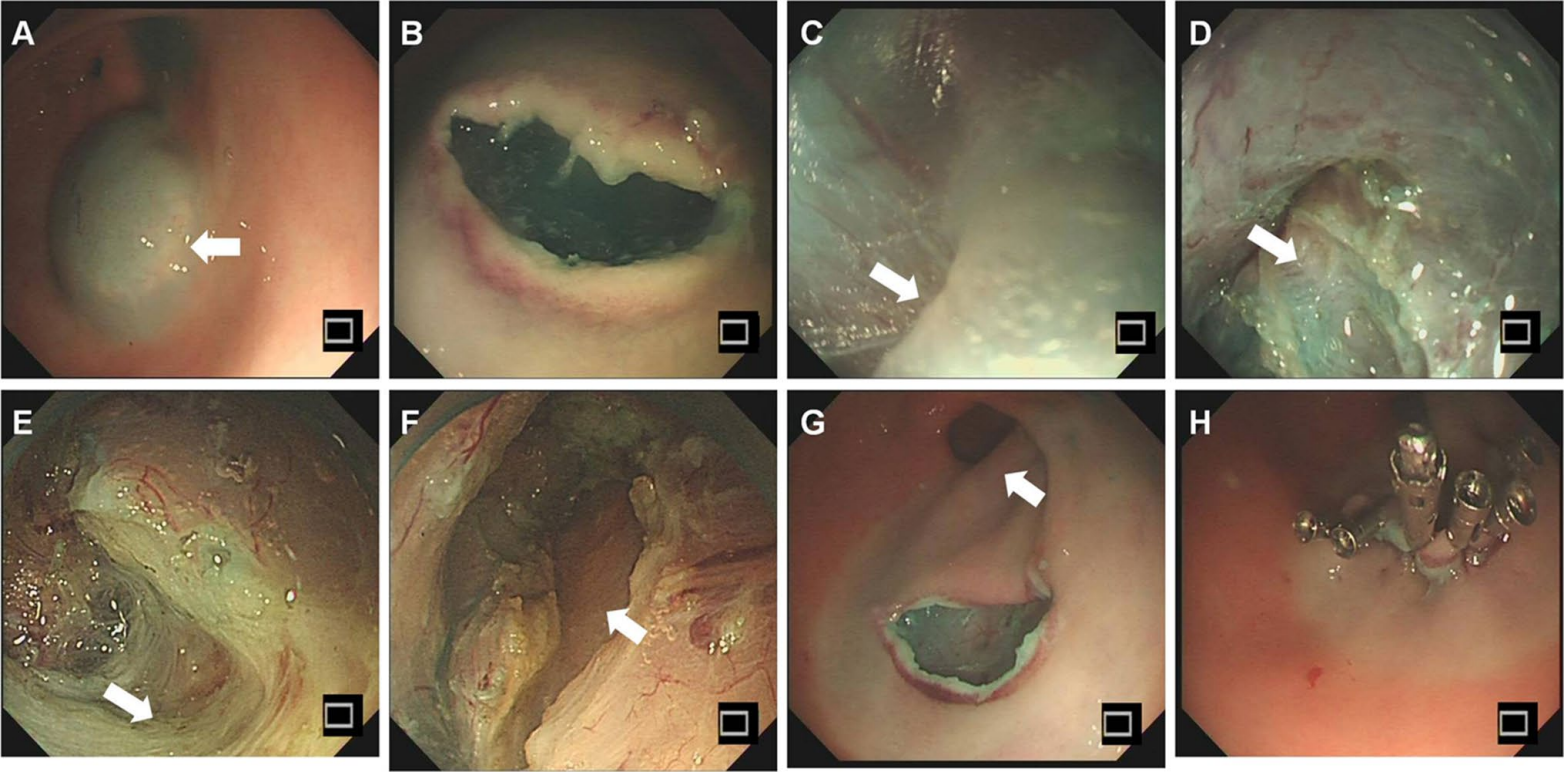
G-POEM procedure. **A** Injection. **B** Submucosal tunnel was established. **C** A white thick pyloric band was in contrast to a thin-walled submucosal space of the duodenum. **D** Dissection of the pyloric ring exposing muscular layer underneath. **E** Circular and longitudinal myotomy for 2–3 cm. **F** Serosa indicates appropriate depth of myotomy. **G** Reevaluation of the mucosotomy site. **H** Mucosal entry was closed with clips from distal mucosal incision point

The G-POEM procedure was performed by a single expert who has profound experience in submucosal endoscopic operation technique. Patients were kept fasting for 12 h prior to G-POEM. Cefoperazone/sulbactam (2.0 g) or levofloxacin (0.5 g) was given intravenously 1 h prior to G-POEM. G-POEM procedure was performed in the operating room with general anesthesia. Blood pressure, electrocardiogram, and pulse oximetry were monitored throughout the procedure. Patients were in left lateral position. A high-definition gastroscope (GIF-HQ 290; Olympus, Tokyo, Japan) with a transparent cap was used. The mucosal incision was made at 5 cm proximal to the pylorus on the gastric greater curvature. Approximately, 10 ml premixed methylene blue/epinephrine/normal saline solution was injected into the mucosa through a sclerotherapy needle (23G, NM4004-04 2, Olympus, Japan). The premixed solution was composed of 500 ml saline mixed with 0.5 ml of 1:1000 epinephrine and 5 ml of methylene blue. A 1.5–2 cm longitudinal mucosal incision was made with a triangular tip knife (KD 640L, Olympus, Tokyo, Japan). Mode of the triangular tip knife was adjusted to Endo Cut I at 60 W on effect 2/cutting width 3/time interval 3 (ERBE, Tubingen, Germany). Then, a submucosal tunnel was made by using the triangular tip knife (KD 640L) with force coag mode at 60 W on effect 2 (ERBE). The submucosal tunnel was extended toward the pylorus and ended at 1 cm beneath the pylorus. Full-thickness antral myotomy was performed by using the triangular tip knife (KD 640L) with force coag mode at 60 W on effect 2 (ERBE). Close attention must be paid to the dissecting orientation to ensure the mucosal layer and serosal layer were unwounded during dissection. Lastly, the mucosal entry was closed with hemostatic clips after the tunnel was rinsed with saline. Low-flow carbon dioxide insufflation was used throughout the procedure. The G-POEM procedure is shown in Fig. 1.

### Statistical analysis

Statistical analyses were conducted by using SPSS software (SPSS 17.0, Chicago, IL). Normally distributed data were expressed as mean (standard deviation). Non-normally distributed data was expressed as median [interquartile range (IQR)]. *T* test was used to compare GCSI between postsurgical gastroparesis patients with different onset times and different courses. Two-way ANOVA were performed to analyze GCSI, GERDQ, half-empty time, and full-empty time in patients with different anastomotic sites before/after G-POEM. Unary linear correlation was used to analyze the relationship between GCSI and pyloric morphologic parameters. *P* values < 0.05 were considered as statistical differences.

## Results

In all the 79 patients, neither pyloric stenosis nor hypertrophy was found before G-POEM. The patient's baseline information is shown in Table 1. All procedures were technically successful. Mean length of myotomy was 2.53 ± 0.52 cm. Mean duration of procedure was 26.35 ± 4.15 min. All mucosal entry points were closed with 3–8 hemostatic clips. Mean postsurgical fasting time was 2.23 ± 0.44 days. Mean length of hospital stay was 5.35 ± 0.82 days.

**Table 1:** The patient's baseline information

	Postsurgical (79)
Age (Y)	65.74 ± 11.11
Sex (F/M)	22/57
BMI	19.74 ± 3.54
Time from surgery to postoperative gastroparesis (Y)	4 (1.5, 10)
≤ 2 years (*n*)	29
> 2 years (*n*)	50
Course of postoperative gastroparesis (Y)	1 (0.5, 6)
≤ 1 year (*n*)	43
> 1 years (*n*)	36
Past surgical history	
Surgery for cardia cancer	35
Surgery for esophageal carcinoma	33
Subtotal gastrectomy for gastric carcinoma	3
Resection for gastric cardiac leiomyoma	5
Repairment for esophageal perforation	1
Subtotal gastrectomy for gastric ulcer	1
Repairment for hiatus hernia	1
Past medical history	
Constipation	13
Diarrhea	3
Hypertension	15
Tachycardia	2
Coronary heart disease	4
Parkinson’s disease	2
Site of anastomosis (the distance from the incisors)	
≤ 20 cm	7
> 20 cm, ≤ 25 cm	15
> 25 cm, ≤ 30 cm	13
> 30 cm, ≤ 40 cm	34
> 40 cm	10

According to the onset time of postsurgical gastroparesis, patients were divided into two groups: gastroparesis occurred within two years after surgery, gastroparesis occurred two years or longer after surgery. The severity of gastroparesis symptoms was not associated with the onset time (GCSI: 2.98 ± 0.64 vs. 2.96 ± 0.59, *p* = 0.551). Based on the course of gastroparesis, patients were divided into two groups: the duration of gastroparesis ≤ 1 year, the duration of gastroparesis > 1 year. The severity of gastroparesis symptoms was not associated with the course of the disease (GCSI: 2.98 ± 0.65 vs. 2.94 ± 0.53, *p* = 0.14).

## The primary outcomes

### The symptomatic improvement based on GCSI

To evaluate the overall clinical effectiveness of G-POEM, GCSI was analyzed before G-POEM and 6-month, 12-month, 18-month, and 24-month post-G-POEM. At 6-month post-G-POEM, 77.2% of patients (61/79) achieved clinical responses with a decrease in mean GCSI from 2.97 ± 0.6 at baseline to 1.12 ± 0.77. At 12-month, 78.3% of patients (47/60) achieved clinical response with a mean GCSI of 1.04 ± 0.53. At 18-month, 74.5% (35/47) of patients achieved clinical responses with a mean GCSI of 1.22 ± 0.58. At 24-month, 81.8% (27/33) of patients achieved clinical responses with a mean GCSI of 0.88 ± 0.39. G-POEM significantly reduced GCSI throughout the follow-up period (*p* < 0.0001 at 6-month, 12-month, 18-month, and 24-month, respectively) (Table 2).

**Table 2:** Overall clinical effectiveness of G-POEM

	Before	6 month	12 month	18 month	24 month
Total (*n*)	79	79	60	47	33
Clinical response (*n*)		61	47	35	27
GCSI	2.97 ± 0.6	1.12 ± 0.77	1.04 ± 0.53	1.22 ± 0.58	0.88 ± 0.39
*p* value*		< 0.0001	< 0.0001	< 0.0001	< 0.0001

*Compared with GCSI before G-POEM

**Fig. 2:**
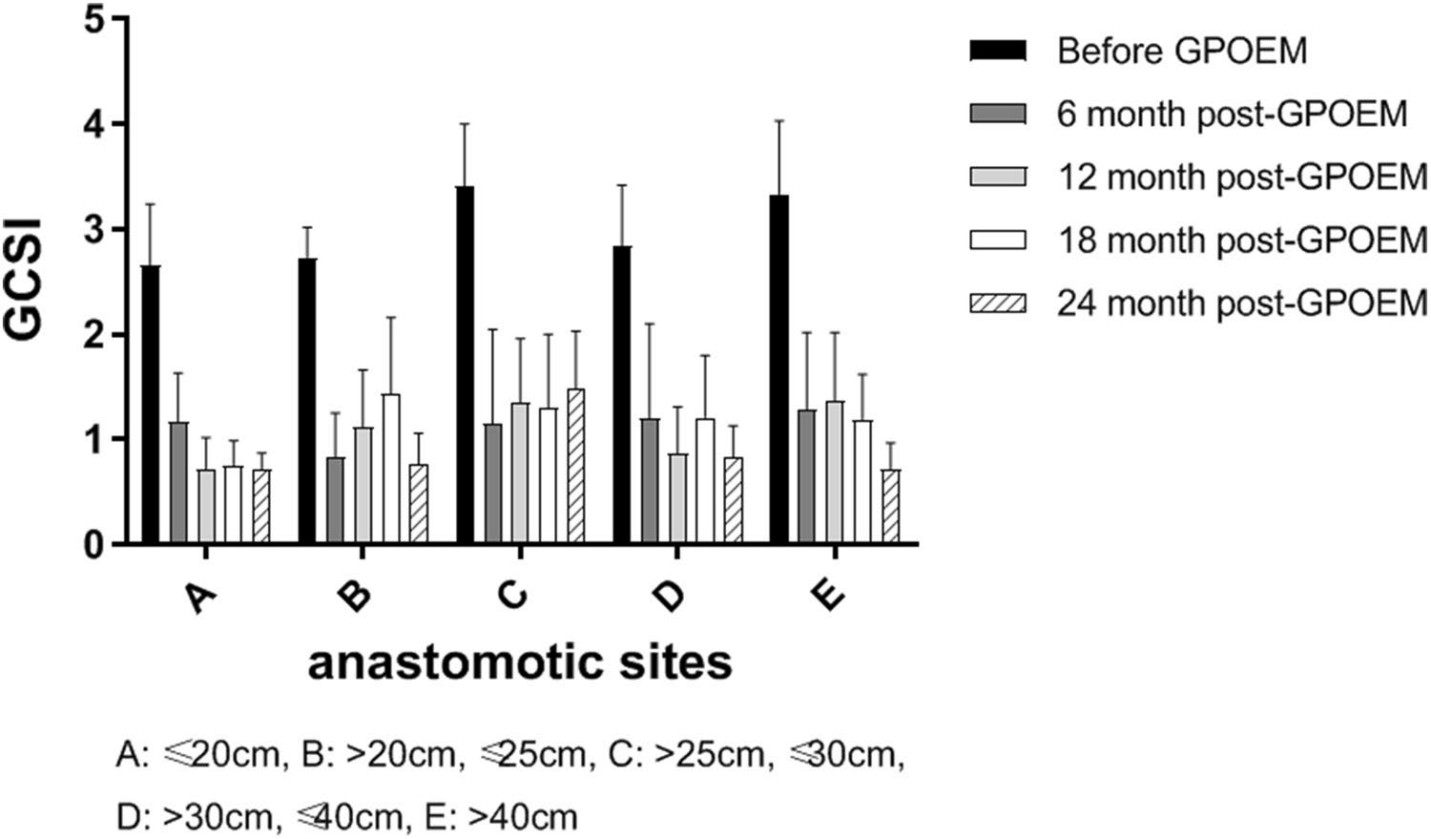
GCSI before and after G-POEM in patients with different anastomotic sites. Before G-POEM, no significant difference of GCSI was found between patients with different anastomotic sites. At 6-month post-G-POEM, a significant improvement of GCSI was found among the patients with different anastomotic sites (*F*_4,165_ = 74.18, *p* < 0.0001)

**Fig. 3:**
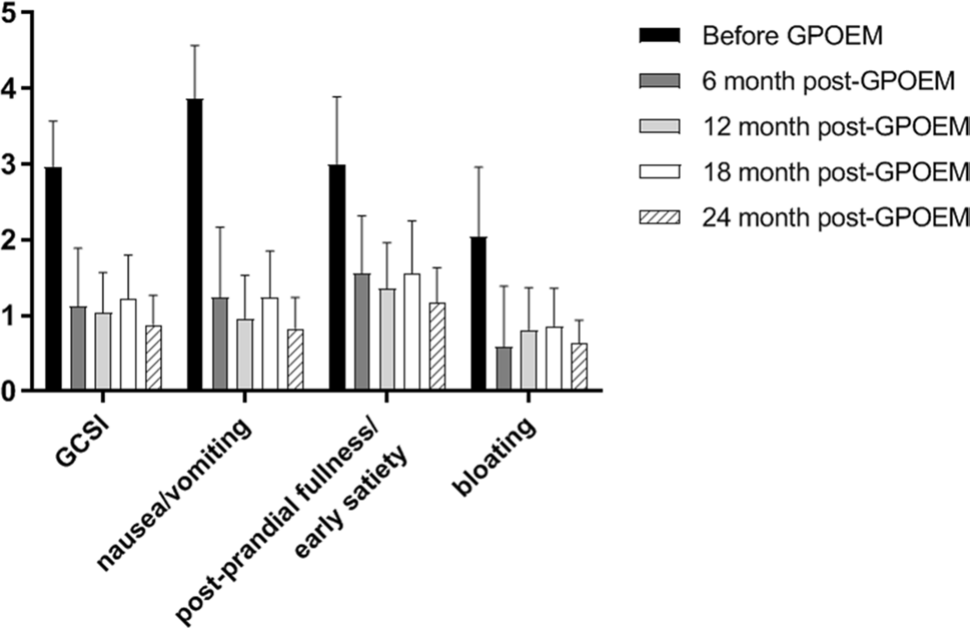
Subscale analysis of GCSI before and after G-POEM. Nausea/vomiting contributed most in pre-G-POEM GCSI. Nausea/vomiting, post-prandial fullness/early satiety, and bloating were improved significantly at 6-month post-G-POEM (*p* < 0.0001, respectively)

For each anastomotic sites, GCSI was evaluated before G-POEM and 6-month, 12-month, 18-month, and 24-month post-G-POEM (Fig. 2). Before G-POEM, no significant difference of GCSI was found between patients with different anastomotic site (Supplementary Table 5). At 6 month post-G-POEM, a significant improvement of GCSI was found among the patients with different anastomotic sites (*F*_4,165_ = 74.18, *p* < 0.0001). The GCSI score was calculated by averaging the mean score of three subscales: nausea/vomiting, post-prandial fullness/early satiety, and bloating. In the present study, nausea/vomiting contributed most in pre-G-POEM GCSI. Subscale analysis demonstrated that nausea/vomiting, post-prandial fullness/early satiety, and bloating were improved significantly at 6 month post-G-POEM (*p* < 0.0001, respectively), which trends were consistent with GCSI (Fig. 3).

### The improvement of gastric emptying function

**Fig. 4:**
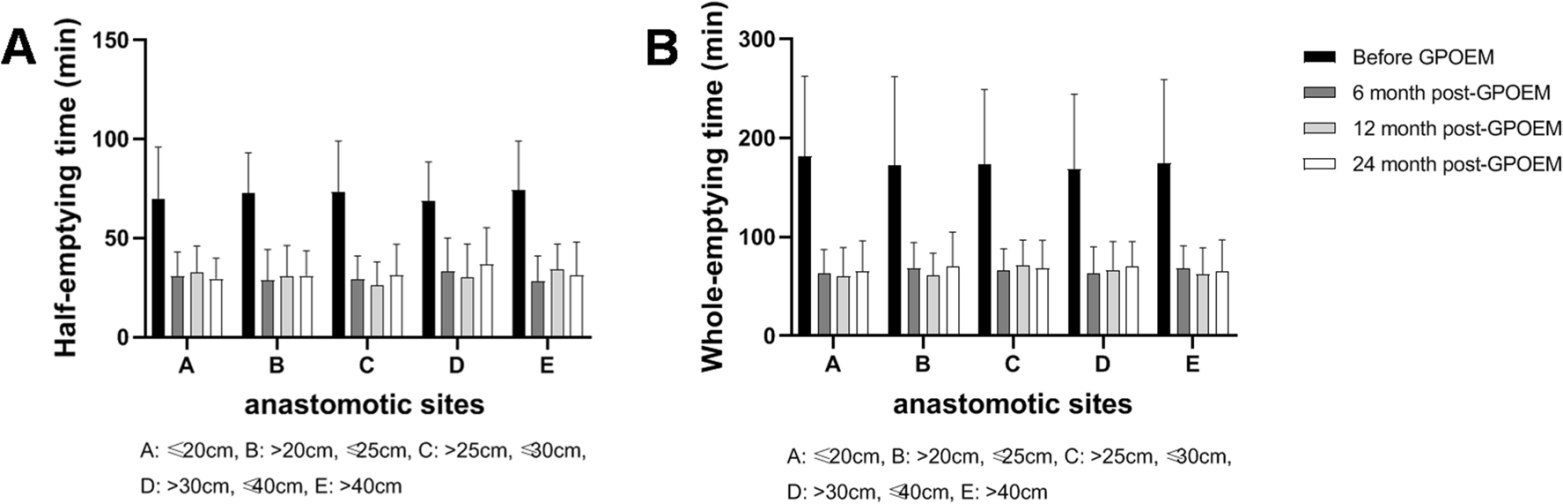
The half-emptying time and whole-emptying time before and after G-POEM. **A** The half-emptying time was significantly shortened in patients with different anastomotic site after G-POEM (*F*_3,174_ = 65.44, *p* < 0.0001). **B** The whole-emptying time was significantly shortened in patients with different anastomotic site after G-POEM (*F*_3,174_ = 54.85, *p* < 0.0001)

**Fig. 5:**
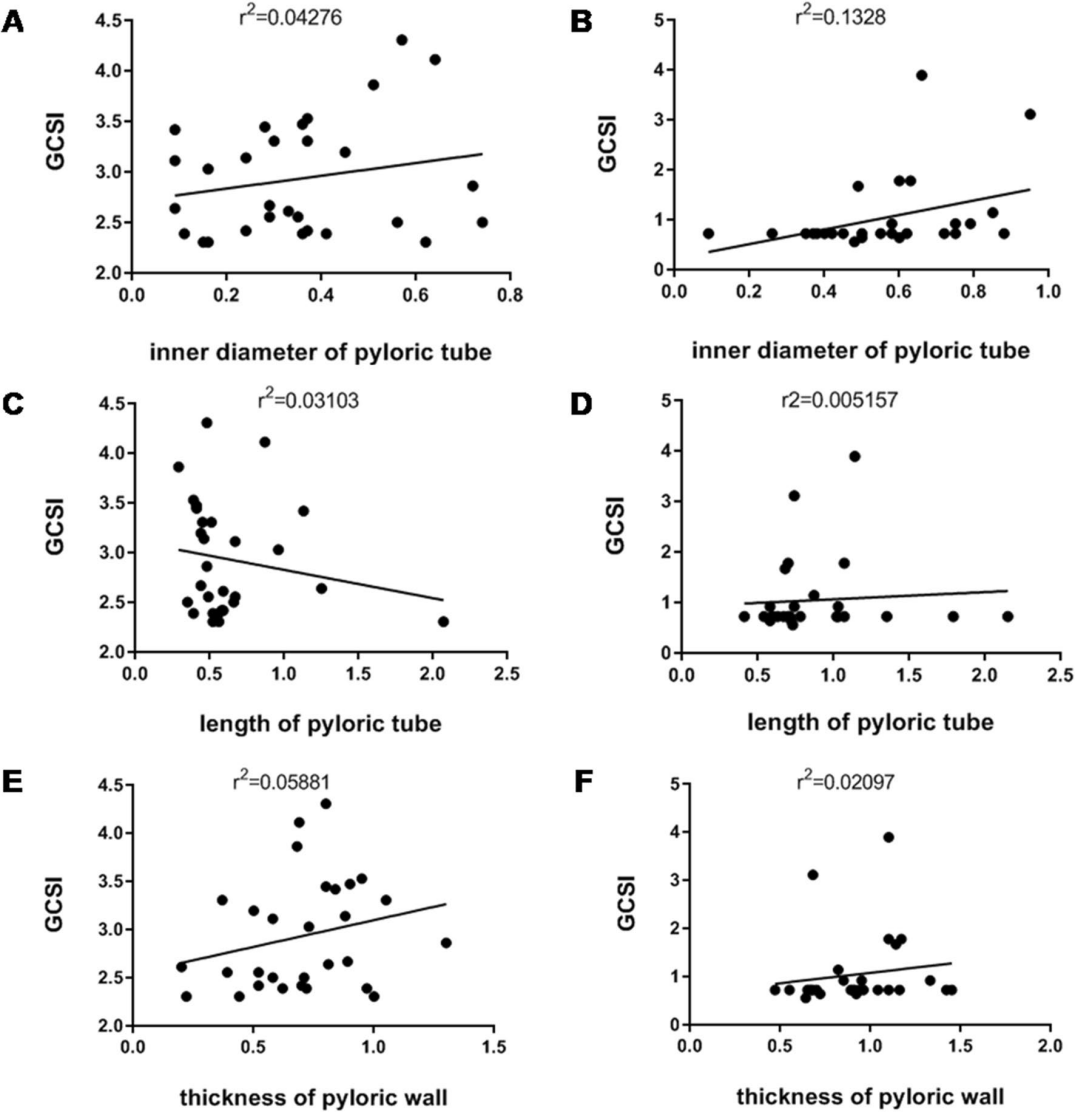
The relationship between GCSI and pyloric morphology. **A**, **B** GCSI wasn't related to the inner diameter of pyloric tube. **C**, **D** GCSI wasn’t related to the length of pyloric tube. **E**, **F** GCSI wasn't related to the thickness of pyloric wall

According to the follow-up protocol, gastric antrum volume was measured using three-dimensional ultrasound examination before G-POEM, 6-month post-G-POEM, 12-month post-G-POEM, and 24 month post-G-POEM. Till 24-month post-G-POEM, a total of 29 patients had completed each three-dimensional ultrasound examination on time. Among them, 27 patients achieved clinical responses based on GCSI. The half-emptying time and whole-emptying time assessed by antral volume alteration were significantly shortened in patients with different anastomotic site after G-POEM (half-emptying time: *F*_3,174_ = 65.44, *p* < 0.0001; whole-emptying time: *F*_3,174_ = 54.85, *p* < 0.0001) (Fig. 4). We also noticed that GCSI wasn't related to the inner diameter of pyloric tube, length of pyloric tube, and thickness of pyloric wall, neither before nor after G-POEM (Fig. 5).

**Fig. 6:**
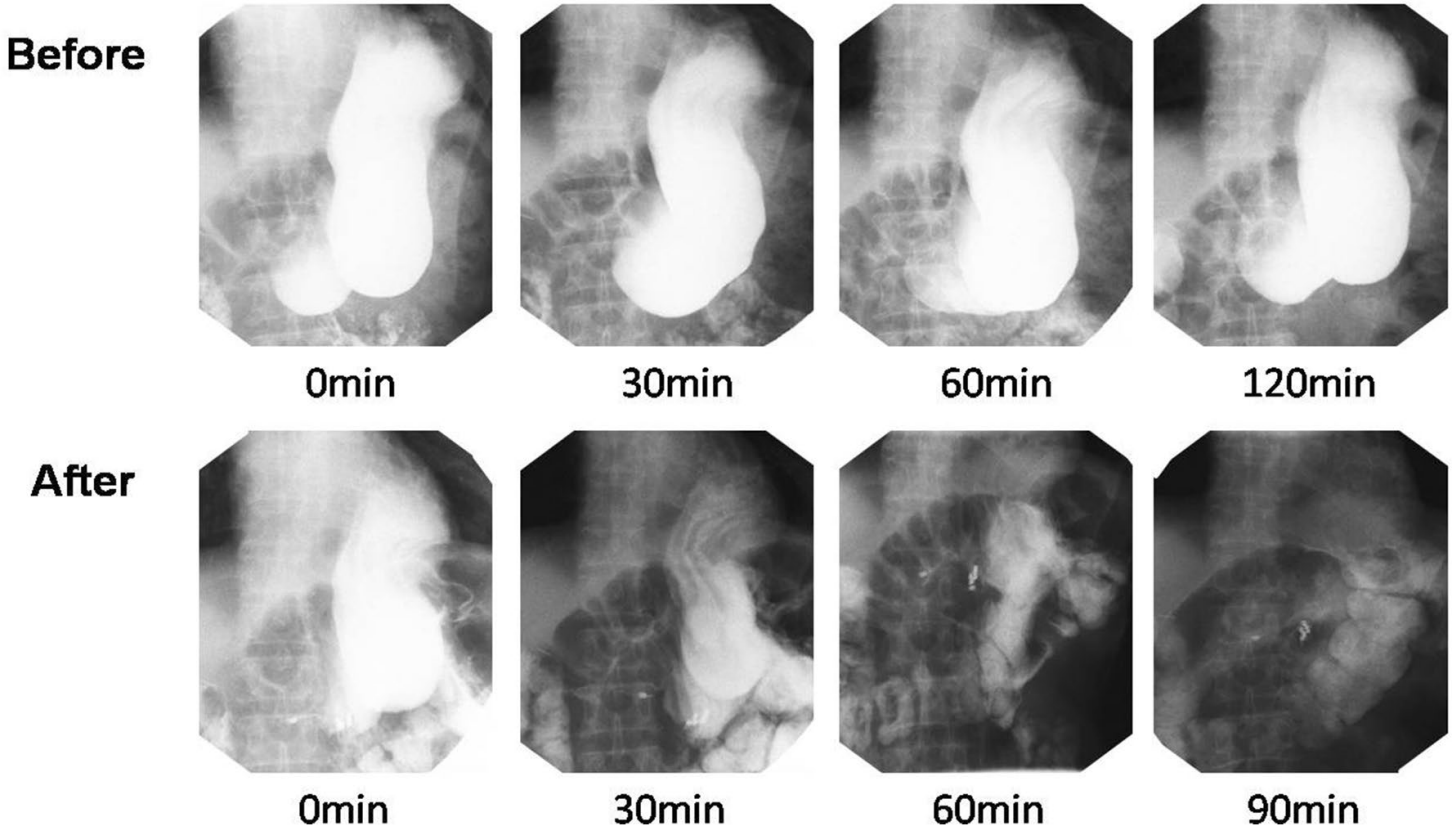
Gastric emptying imaging before and after G-POEM. The emptying rate of ioversol was obviously accelerated after G-POEM

Gastric emptying imaging was taken in all the patients before G-POEM, 6-month post-G-POEM, 12-month post-G-POEM, and 24-month post-G-POEM. Although there was no statistical data, the emptying of ioversol was observed accelerated in all the patients after G-POEM (Fig. 6).

## The secondary outcomes

### The improvement of gastroesophageal reflux symptoms

**Fig. 7:**
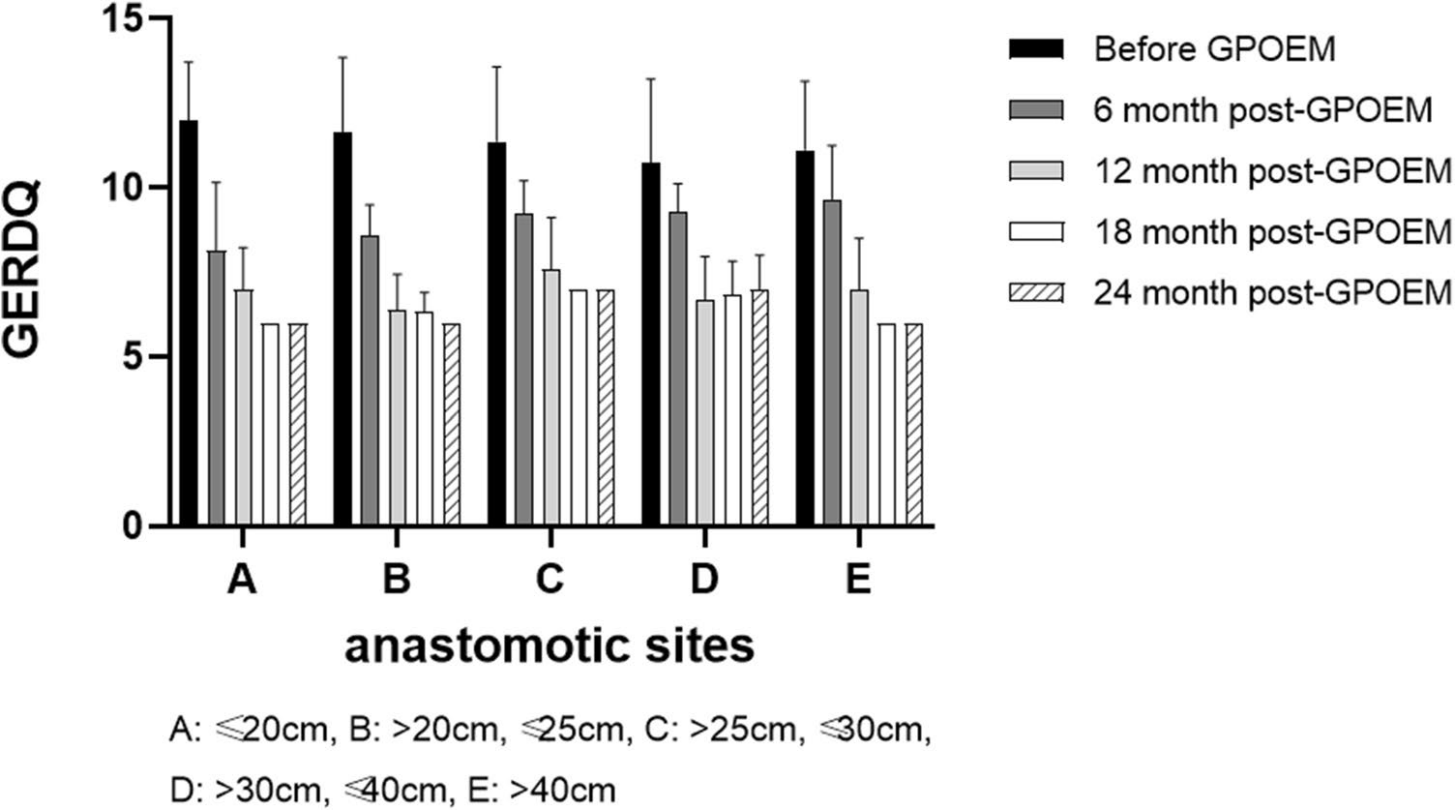
GERDQ was supplemented to evaluate the clinical efficacy of G-POEM. For each time points (pre-G-POEM or post-G-POEM), GERDQ didn't differ statistically significantly in patients with different anastomotic site (*F*_4,118_ = 0.7391, *p* = 0.5672). For each anastomotic site, GERDQ improved significantly at different time points (*F*_4,118_ = 63.47, *p* < 0.0001). The higher the esophageal anastomotic site was, the faster G-POEM improved the symptoms of gastroesophageal reflux

Thirty-one patients (31/79, 39.2%) also suffered from symptoms of gastroesophageal reflux before G-POEM. Therefore, GERDQ was also used to evaluate the clinical efficacy of G-POEM (Fig. 7). For each time points (pre-G-POEM or post-G-POEM), GERDQ didn't differ statistically significantly in patients with different anastomotic site (*F*_4,104_ = 0.8075, *p* = 0.5231). For each anastomotic site, GERDQ was improved significantly at different time points (*F*_4,104_ = 59.11, *p* < 0.0001) (Table 3). In patients with high esophageal anastomotic site (distance between the anastomosis and the incisors ≤ 20 cm or > 20 cm, ≤ 25 cm), GERDQ was improved significantly at 6 month post-G-POEM (*p* < 0.0001, respectively). In patients with middle or low anastomotic site (distance between the anastomosis and the incisors > 25 cm), GERDQ was improved significantly at 12-month post-G-POEM (*p* < 0.0001, respectively). Additionally, we found that most of the patients who didn't achieve clinical responses (based on GCSI) had symptoms of gastroesophageal reflux before G-POEM (Supplementary Table 6).

**Table 3:** GERDQ before and after G-POEM in patients with different anastomotic sites

Anastomotic site	Time point	GERDQ	*p* value
≤ 20 cm			
	Before vs. 6 month	12.0 ± 1.7 vs. 8.2 ± 2.0	0.0093
	Before vs. 12 month	12.0 ± 1.7 vs. 7.0 ± 1.2	0.0002
	Before vs. 18 month	12.0 ± 1.7 vs. 6.0	< 0.0001
	Before vs. 24 month	12.0 ± 1.7 vs. 6.0	< 0.0001
> 20 cm, ≤ 25 cm			
	Before vs. 6 month	11.7 ± 2.2 vs. 8.6 ± 0.9	0.0082
	Before vs. 12 month	11.7 ± 2.2 vs. 6.4 ± 1.0	< 0.0001
	Before vs. 18 month	11.7 ± 2.2 vs. 6.3 ± 0.6	< 0.0001
	Before vs. 24 month	11.7 ± 2.2 vs. 6.0	< 0.0001
> 25 cm, ≤ 30 cm			
	Before vs. 6 month	11.4 ± 2.2 vs. 9.3 ± 1.0	0.7829
	Before vs. 12 month	11.4 ± 2.2 vs. 7.6 ± 1.5	0.0122
	Before vs. 18 month	11.4 ± 2.2 vs. 7.0	0.0030
	Before vs. 24 month	11.4 ± 2.2 vs. 7.0	0.0030
> 30 cm, ≤ 40 cm			
	Before vs. 6 month	10.7 ± 2.5 vs. 9.3 ± 0.8	0.9444
	Before vs. 12 month	10.7 ± 2.5 vs. 6.7 ± 1.3	0.0002
	Before vs. 18 month	10.7 ± 2.5 vs. 6.8 ± 1.0	0.0002
	Before vs. 24 month	10.7 ± 2.5 vs. 7.0 ± 1.0	0.0029
> 40 cm			
	Before *vs.* 6 month	11.1 ± 2.0 vs. 9.7 ± 1.6	0.9955
	Before vs. 12 month	11.1 ± 2.0 vs. 7.0 ± 1.5	0.0030
	Before vs. 18 month	11.1 ± 2.0 vs. 6.0	0.0001
	Before vs. 24 month	11.1 ± 2.0 vs. 6.0	0.0008

### Adverse events and complications of G-POEM

G-POEM procedure was successfully performed in all the patients (100%) without any adverse event.

Seven patients (8.8%) suffered upper abdominal pain (NRS score ≤ 3) on the first day after G-POEM. Anti-inflammatory and analgesic treatments were given when perforation and peritonitis were excluded. Then abdominal pain relieved without recurrence. All the patients were given intravenous nutritional support after G-POEM. No one suffered from dehydration, electrolyte abnormality, and malnutrition. After hospital discharge, no one required re-hospitalization for any complications or discomfort.

### Outcomes of body mass index, medications, and diet status

BMI improved from 19.74 ± 3.54 before G-POEM to 21.70 ± 3.45 at 24 months post-G-POEM (*p* = 0.000). Compared to daily use (79/79, 100%) before G-POEM, 27 patients (27/33, 81.8%) with clinical response (based on GCSI) were able to discontinue prokinetic agents, whereas 6 (6/33, 18.2%) took prokinetic agents as needed. Thirty-one patients (31/79, 39.2%) used proton pump inhibitor (PPI) daily before G-POEM. At 24-month post-G-POEM, only 2 patients (2/33, 6%) took PPI as needed.

After G-POEM procedure, all the patients were asked for hospitalization and fasting over 48 h. Antibiotics (cefoperazone/sulbactam or levofloxacin) and proton pump inhibitor were administered intravenously. After 48 h post-procedure, a liquid diet could be given to patients who didn't have any discomfort or complication. Patients were then discharged if they could eat liquid diet without vomiting and abdominal pain. After one-week post-procedure, a semi-liquid and low-fiber diet was given to those patients who had tolerated liquid diet. Normal diet was restored in those who had tolerated semi-liquid diet till one-month post-procedure.

## Discussion

Postsurgical gastroparesis is a complication after esophageal and gastrointestinal surgery and other surgical operations affecting the upper digestive tract. The main clinical manifestations are nausea, vomiting, upper abdominal fullness, belching, gastric retention, and weight loss. At present, the mechanism of postsurgical gastroparesis is still unclear. The possible pathogenesis is listed as follows [2, 25–28]: 1. the physiological shape, size, position, and positive pressure environment of the residual stomach are changed, leading to the decrease of pressure gradient in the stomach and duodenum; 2. surgery usually excise or injure the vagus nerve trunk, resulting in decreased or disappeared gastric tension and peristalsis, as well as pyloric dysfunction; 3. prolonged pulling and kneading during the operation will cause damage of the gastric wall, then reduce the tension and peristalsis of the residual stomach; 4. reduced blood supply of the residual gastric leads to the inhibition of gastrointestinal motility; and 5. the environment of G cells in the residual stomach are changed, leading to increased gastrin secretion.According to the onset time, postsurgical gastroparesis can be divided into acute and chronic [29]. Acute postsurgical gastroparesis occurs within 1–2 days after commencing diet, or when the diet changes from fluid to semi-fluid. Most cases of acute postoperative gastroparesis can be relieved by conservative medical treatment. Chronic postsurgical gastroparesis occurs within weeks, months or even years after surgery, seriously affecting the quality of patients' life.

Conservative treatments were often ineffective to relieve symptoms of chronic postsurgical gastroparesis. Operations were increasingly performed to therapy patients with postsurgical gastroparesis. Combined the effectiveness of pyloromyotomy with the potential benefits of minimally invasive endoscopic technique, the technology of G-POEM was invented. In 2012, the first G-POEM procedure was successful in a porcine model [30]. The first clinical application was successful in the following year [10]. After that, G-POEM was gradually used to treat refractory gastroparesis [10–17, 31–35].

As we know, there was rare study published on the outcome of G-POEM in postsurgical gastroparesis [36]. The present study provided 24-month data, which was the longest follow-up of G-POEM in treating postsurgical gastroparesis. To explore the possible correlation factors of the severity of gastroparesis symptoms, we analyzed GCSI before G-POEM. The severity of gastroparesis symptoms wasn't related with the onset time postoperation, nor the time course of gastroparesis. No relationship was found between the severity of gastroparesis symptoms and the site of anastomosis. Subscales of GCSI was consisted of nausea/vomiting, post-prandial fullness/early satiety and bloating. In the present study, scores of nausea/vomiting subscale contributed most in pre-G-POEM GCSI. This was consistent with the point that certain cardinal symptoms correlate with certain pathophysiological mechanism of gastroparesis [14]. Consisted of preoperative examination and postoperative hospital stay, the total hospital stay seemed quite long in our research. Therefore, we used postoperative fasting time to reflect the postoperative recovery. Mean postsurgical fasting time (2.23 ± 0.44 days) was similar to the postoperative recovery of other studies (often represented by hospital stay) [10–17, 31–35].

To explore the effect of G-POEM in improving the symptoms of postsurgical gastroparesis, we analyzed GCSI at 6-month, 12-month, 18-month, and 24-month post-G-POEM, for different anastomotic sites. G-POEM significantly improved the symptoms of gastroparesis throughout the follow-up period. Scores of GCSI subscales were all reduced throughout the follow-up period, which meant the symptoms of nausea/vomiting, post-prandial fullness/early satiety, and bloating were all improved. Thirty-one patients (31/79, 39.2%) suffered from symptoms of gastroesophageal reflux before G-POEM. Therefore, GERDQ was also used to evaluate the clinical efficacy of G-POEM. After G-POEM, GERDQ was improved significantly at different time points in patients with different anastomotic sites. The higher the esophageal anastomotic site was, the faster G-POEM improved the symptoms of GERD. We also found most of the patients who didn't achieve clinical responses (based on GCSI) had symptoms of gastroesophageal reflux before G-POEM. The above suggested GERDQ may be considered as an additional method to evaluate the clinical efficacy of G-POEM. The half-emptying time and whole-emptying time assessed by antral volume alteration were significantly shortened after G-POEM in patients with different anastomotic site. Gastric emptying imaging showed the emptying of ioversol was observed accelerated obviously after G-POEM. These findings supported that G-POEM, which targeted the pylorus, could improve symptoms of postsurgical gastroparesis and gastric emptying function of residual stomach. However, it was similar to the reported literature [2, 37, 38] that no positive correlation between the improvement of symptoms and gastric emptying was noted.

Until now, no consensus was reached in the optimal depth and length of myotomy [10, 39]. We performed the full-thickness myotomy in the present research. As we know, the procedure of G-POEM was based on POEM. Simple circular myotomy didn't always lead to satisfactory outcome in studies of POEM for achalasia, but full-thickness myotomy could cover the shortage of circular myotomy [40–43]. Additionally, completeness of myotomy was also considered the prerequisite for excellent long-term results of conventional surgical myotomy [42, 44, 45]. According to the muscle anatomy, the longitudinal muscle closes to the circular muscle and has weak connection with the serous membrane. Full-thickness myotomy could decrease the procedure time because the time consumed by carefully distinguishing and protecting the longitudinal muscle was saved [43]. Till now, no data showed full-thickness myotomy could increase the procedure-related adverse events. However, further researches are still needed to compare selective circular myotomy and full-thickness myotomy in G-POEM. It's known that the duodenal bulb is prone to perforation and bleeding due to its thin wall and rich blood circulation. Therefore, we located the distal point of submucosal tunnel at 1 cm beneath the pylorus, trying to avoid perforation or bleeding while decreasing pyloric channel pressure. Some experts considered that a long (> 2 cm) antral myotomy may make the antral contractility and gastric motility be worsen [10]. However, others considered that 2.5–3 cm myotomy was safe and effective [14, 39]. In our study, the mean length of antral myotomy was 2.53 ± 0.52 cm. No adverse events occurred.

Scintigraphy was considered as the ‘gold standard’ technique to measure gastric emptying in both clinical and research. Nevertheless, the application of scintigraphy, particularly in pregnant women and children, was restricted by exposure to ionizing radiation. Because of the specialized and expensive equipment, scintigraphy was relatively costly and not always readily available. In the present research, three-dimensional ultrasonography and gastric emptying imaging were used to measure gastric emptying. Three-dimensional ultrasonography had been appeared to be an accurate and valid measurement of gastric emptying, which had good correlation and consistency with scintigraphy [18–23]. Three-dimensional ultrasonography made observation of the gastric morphology and dynamics become more convenient, which could facilitate further research in postsurgical gastroparesis. In the present study, we noticed that GCSI wasn't related to pyloric length, pyloric diameter,, and thickness of pyloric wall. More work should be done to explore the potential relationship between GCSI and manometric measurements of residual gastric antrum.

The present study had several limitations. Firstly, this was a single-center study. Although this study presented the largest number of patients and the longest follow-up time in postsurgical gastroparesis, the number of patients was still small. Not all the patients who were included in the study had reached the 24-month follow-up period yet. Secondly, the direction and frequency of pyloric contraction, pyloric distensibility, and transpyloric pressure were thought to be closely related to the therapeutic effect. However, we didn't measure these parameters for the lack of consensus in the previous studies [46, 47]. Thirdly, the G-POEM procedure was performed by only one highly experienced endoscopist. The procedure may be not widely performed by the general endoscopists, so the authority of the procedural techniques and the repeatability of the results still need to be tested. Fourthly, to assess the therapeutic effect of G-POEM, we evaluated the improvement in symptom and measured the gastric emptying function. However, there could be more methods to assess the improvement in the health related quality of life, for example, the short-form 36-item health survey questionnaire (SF-36).

The result of the present study is positive, which means that G-POEM can be considered as an effective and less-invasive treatment for postsurgical gastroparesis. But the mechanism that G-POEM improves or normalizes gastric emptying is still unknown. Manometric measurements would be an exciting topic in the further research about the physiologic mechanism of G-POEM.

## Electronic supplementary material

Below is the link to the electronic supplementary material.

Supplementary file1 (DOCX 21 kb)
