# Supplementary material for: Feasibility, safety, and long-term efficacy of gastric peroral endoscopic myotomy (G-POEM) for postsurgical gastroparesis: a single-center and retrospective study of a prospective database
Source: Surg Endosc. 2020 Sep 3;35(7):3459–70. doi: 10.1007/s00464-020-07793-0 (PMC8195960; doi:10.1007/s00464-020-07793-0)
Supplement: Supplementary file 1 — Supplementary file1 (DOCX 21 kb) [file 464_2020_7793_MOESM1_ESM.docx]

**Supplementary Table 1**

**Medical management before the study**

| Drugs | Number | medication Duration (Y) |
| --- | --- | --- |
| Prokinetic agents | 79/79, 100% |  |
| mosapride | 36 | 1.4±0.6 |
| cisapride | 6 | 1.2±0.8 |
| domperidone | 8 | 1±0.5 |
| proprietary chinese medicine* | 38 | 2.5±1.5 |
| Proton pump inhibitor | 31/79, 39.2% |  |
| rabeprazole | 11 | 1.6±0.4 |
| lansoprazole | 9 | 1.2±0.6 |
| omeprazole | 8 | 1.4±0.8 |
| esomeprazol | 3 | 1.2±0.6 |

*Some patients take both proprietary Chinese medicine and other prokinetic agents

**Supplementary Table 2**

**Gastroparesis Cardinal Symptoms Index (GCSI) scores.**

| **Symptoms** | **None** | **Very mild** | **Mild** | **Moderate** | **Severe** | **Very severe** |
| --- | --- | --- | --- | --- | --- | --- |
| Nausea | 0 | 1 | 2 | 3 | 4 | 5 |
| Retching | 0 | 1 | 2 | 3 | 4 | 5 |
| Vomiting | 0 | 1 | 2 | 3 | 4 | 5 |
| Stomach fullness | 0 | 1 | 2 | 3 | 4 | 5 |
| Early satiety | 0 | 1 | 2 | 3 | 4 | 5 |
| Excessive postprandial fullness | 0 | 1 | 2 | 3 | 4 | 5 |
| Loss of appetite | 0 | 1 | 2 | 3 | 4 | 5 |
| Bloating | 0 | 1 | 2 | 3 | 4 | 5 |
| Visibly distended abdomen | 0 | 1 | 2 | 3 | 4 | 5 |

The patients were asked to describe an average severity of each symptom. The GCSI score was calculated by averaging the mean score of 3 subscales: nausea/vomiting, post-prandial fullness/early satiety and bloating. Patients with GCSI score ≥ 2.3 are considered to be suffered from gastroparesis.

**Supplementary Table 3**

**Gastroesophageal Reflux Disease Questionnaire (GERDQ) scores[43].**

| **Question** | **Frequency score (points) for symptom** | | | |
| --- | --- | --- | --- | --- |
|  | **0 day** | **1 day** | **2-3 day** | **4-7 day** |
| 1. How often did you have a burning feeling behind your breastbone (heartburn)? | 0 | 1 | 2 | 3 |
| 2. How often did you have stomach contents (liquid or food) moving upwards to your throat or mouth (regurgitation)? | 0 | 1 | 2 | 3 |
| 3. How often did you have pain in the centre of the upper stomach? | 3 | 2 | 1 | 0 |
| 4. How often did you have nausea? | 3 | 2 | 1 | 0 |
| 5. How often did you have difficulty getting a good night's sleep because of your heartburn and/or regurgitation? | 0 | 1 | 2 | 3 |
| 6. How often did you take additional medication for your heartburn and/or regurgitation, other than what the physician told you to take) (such as Tums, Rolaids, Maalox?) | 0 | 1 | 2 | 3 |

Patients are asked to score the number of days with symptoms and use of over-the-counter (OTC) medications during the previous 7 days. The frequency of four positive predictors of GERD (heartburn, regurgitation, sleep disturbance due to reflux symptoms or use of over-the-counter (OTC) medications for reflux symptoms) are scored by a four graded Likert scale (0–3). Two negative predictors of GERD (epigastric pain and nausea) are scored by a reversed Likert scale (3–0). A total GerdQ score ranges of 0–18.

**Supplementary Table 4**

**The post-procedural time frame.**

|  | Baseline | 6 month | 12months | 18 months | 24 months |
| --- | --- | --- | --- | --- | --- |
| GCSI | √ | √ | √ | √ | √ |
| GERDQ | √ | √ | √ | √ | √ |
| Upper endoscopy | √ | √ | √ | - | √ |
| 3D ultrasonography | √ | √ | √ | - | √ |
| gastric emptying imaging | √ | √ | √ | - | √ |

**Supplementary Table 5**

**GCSI between patients with different anastomotic before GPOEM.**

| anastomotic site | GCSI | p value |
| --- | --- | --- |
| ≤20 *vs.* >20, ≤25 | 2.66 ± 0.58 *vs* 2.73 ± 0.29 | 0.9997 |
| ≤20 *vs.* >25, ≤30 | 2.66 ± 0.58 *vs* 3.41± 0.59 | 0.3405 |
| ≤20 *vs.* >30, ≤40 | 2.66 ± 0.58 *vs* 2.84 ± 0.58 | 0.9872 |
| ≤20 *vs.* >40 | 2.66 ± 0.58 *vs* 3.33 ± 0.7 | 0.4410 |
| >20, ≤25 *vs*. >25, ≤30 | 2.73 ± 0.29 *vs* 3.41± 0.59 | 0.1381 |
| >20, ≤25 *vs*. >30, ≤40 | 2.73 ± 0.29 *vs* 2.84 ± 0.58 | 0.9889 |
| >20, ≤25 *vs*. >40 | 2.73 ± 0.29 *vs* 3.33 ± 0.7 | 0.2128 |
| >25, ≤30 *vs*. >30, ≤40 | 3.41± 0.59 *vs* 2.84 ± 0.58 | 0.1894 |
| >25, ≤30 *vs*. >40 | 3.41± 0.59 *vs* 3.33 ± 0.7 | 0.9990 |

**Supplementary Table 6**

**Patients with symptoms of gastroesophageal reflux before GPOEM.**

|  |  | Before | 6 month | 12 month | 18 month | 24 month |
| --- | --- | --- | --- | --- | --- | --- |
| Postsurgical gastroparesis |  | 79 | 79 | 60 | 47 | 33 |
| GER |  | 31 | 31 | 25 | 23 | 19 |
| Clinical response (GCSI) |  |  | 61 | 47 | 35 | 27 |
|  | GER before GPOEM |  | 19 | 16 | 15 | 13 |
| No clinical response (GCSI) |  |  | 18 | 13 | 12 | 6 |
|  | GER before GPOEM |  | 12 | 9 | 8 | 6 |

GER: gastroesophageal reflux
